# Evaluating the photocatalytic efficiency of the BiVO_4_/rGO photocatalyst

**DOI:** 10.1038/s41598-019-52589-5

**Published:** 2019-11-06

**Authors:** Sukon Phanichphant, Auppatham Nakaruk, Kantapat Chansaenpak, Duangdao Channei

**Affiliations:** 1 Center of excellence in Materials Science and Technology, Chiang Mai University, Chiang Mai, 50200 Thailand; 2 Department of Industrial Engineering, Faculty of Engineering, Naresuan University, Phitsanulok, 65000 Thailand; 3 National Nanotechnology Center, National Science and Technology Development Agency, Thailand Science Park, Pathum Thani, 12120 Thailand; 4 Department of Chemistry, Faculty of Science, Naresuan University, Phitsanulok, 65000 Thailand

**Keywords:** Materials science, Nanoscience and technology

## Abstract

The present study reported the preparation of BiVO_4_ by co-precipitation method. The as-prepared BiVO_4_ photocatalyst were deposited on rGO sheets to form BiVO_4_/rGO via the hydrothermal method. The crystalline structure, morphological, optical properties, and surface properties of the synthesized pure BiVO_4_ compared to BiVO_4_/rGO composite were studied using X-ray diffraction (XRD), scanning electronmicroscopy (SEM), photoluminescence (PL) spectrophotoscopy, UV–vis spectrophotometer with an integrating sphere, and N_2_ adsorption-desorption isotherm based on BET theory. The photocatalytic activity of the prepared samples were evaluated by the degradation of MB dye in aqueous medium under visible light irradiation. The result showed that the BiVO_4_/rGO composite exhibited greater photocatalytic efficiency compared to pure BiVO_4_ with the photocatalytic degradation efficiency remains stable up to fifth cycle. The improved activity of the BiVO_4_/rGO composite might be attributed to the high surface area available to adsorb more MB molecules, and efficient charge separation of BiVO_4_ through π electron on the rGO structure. According to experimental results, the possible photocatalytic mechanism of the BiVO_4_/rGO composite were determined and the active species hydroxyl radical were reported. Based on photocatalytic activity inhibition in the presence of both h^+^ (VB) and O_2_^•*−*^ (CB) scavengers over the BiVO_4_ photocatalyst, it can be proposed that the hydroxyl radical generated during the photocatalytic degradation mechanism is mainly responsible by the main active species of h^+^ and O_2_^•*−*^ at VB and CB positions, respectively.

## Introduction

Water resource pollution is of utmost concern by scientist globally due to the increasing demand of water for socioeconomic development and human health. As a result, the over use of water together with water pollution and climate change are the major reasons for water scarcity^1^. Among organic pollutant compound, the organicazo dyes and aromatic organics are normally used in all industries to color the products in order to make them attractive^2^. The removal of colors from wastewater is important owing to their negative effects on the environmental water quality even at low quantities of organic dyes. As a result of their stability in chemical structure, it is very difficult to biodegrade aromatic molecules naturally. Several conventional technologies have been used in recent years for organic contaminant treatment. Among them are some technologies such as adsorption, coagulation, membrane filtration and sedimentation, which can only change organic contaminants from the primary toxic pollutants to secondary pollutants in treatment process rather than degrade those substances completely^3–6^. In addition, they are not effective to meet certain criteria requirements or generate non-biodegradable organic pollutants appearing in effluents after a long period of time. Hence, alternative methods have been developed to solve this issue. Advanced oxidation process (AOPs) are alternative methods that have received much attention for the removal of organic contaminant such as pesticides, organic dyes and industrial organic wastes. For AOPs in wastewater treatment, the highly reactive hydroxyl radicals (OH^•^) are the most powerful oxidizing species in the oxidative reaction of organic pollutant^7,8^. Among AOPs, the heterogeneous photocatalysis based on semiconductor catalyst has established its efficiency in degrading organic compounds and mineralizing them to carbon dioxide, water, and other small fragments^9^. The basic principle of photocatalysis is a process in which the catalyst is excited by light and produces highly oxidizing free radicals such as OH^•^. Pollutants in wastewater are then adsorbed and degraded by these free radicals on the surface of catalyst until they transform into carbon dioxide, water, and non-toxic small fragments. The type of semiconductor catalyst plays an important role in the photocatalytic process such as TiO_2_, CeO_2_, ZnO, WO_3_, BiVO_4_ photocatalyst which are often applied in photocatalytic treatment^10–13^. Among these catalysts, bismuth vanadate (BiVO_4_) has recently attracted considerable attention due to its high photocatalytic activity under visible-light irradiation and its small band gap of ~2.4 eV^14–19^. Although, BiVO_4_ is widely used in the photocatalytic degradation of organic contaminants, the low photocatalytic activity of pure BiVO_4_ is unavoidable due to its poor adsorption performance and the difficulty in migration and separation of electron-hole pairs.

Many researchers have modified BiVO_4_ photocatalysts by metal doping and coupling with other semiconductors in order to enhance charge separation as well as increase the photocatalytic activity. Some research works have been found to combine photocatalyst with reduced graphene oxide (rGO) for the photocatalytic degradation of organic dyes^20–29^. Since the rGO has shown high performance in many applications due to its excellent charge separation ability between the intrinsic delocalized π–π electron, the rGO could promote electron transport between the composite BiVO/rGO photocatalyst and the organic pollutant molecules^30,31^. Because of its high surface area, rGO can be applied as a supporting material, not only to significantly increase the surface area of the system, but also to increase the number of surface active sites. As a result, BiVO_4_/rGO composite particles are able to disperse and stabilize on a very high specific surface area of rGO for potential applications in photocatalysis^32,33^.

Therefore, the aim of this study is to synthesize a multifunctional material of BiVO_4_/rGO composite, combined with the photocatalytic activity of BiVO_4_ coupled with the adsorption and trapping abilities produced from rGO. The synthesized multifunctional material was also applied in the removal of methylene blue (MB). In addition, the analytical techniques including XRD, SEM, PL, DRS, and BET of BiVO_4_ compared to BiVO_4_/rGO composite were analyzed and discussed. Finally, the main radicals participating in the photocatalytic process was conducted by a trapping experiment and the photocatalytic mechanism of BiVO_4_ compared to BiVO_4_/rGO composite was further discussed.

## Experimental

### Preparation of BiVO_4_ photocatalyst by co-precipitation method

Firstly, BiVO_4_ powders was prepared by dissolving bismuth (III) nitrate, and ammonium vanadate in 3 M nitric acid under continuous stirring. The resulting dispersions were separated by centrifugation, washed with deionized water until the pH became neutral, dried at 70 °C for 24 h, and calcined at 550 °C for 4 h.

### Preparation of BiVO_4_/rGO composite by hydrothermal method

Reduced graphene oxide (rGO) powders was synthesized from natural graphite powder by chemical oxidation using the synthesis methods from our previous work^34^. Firstly, the as-prepared rGO powders was added into the mixed solution of bismuth (III) nitrate, ammonium vanadate in 3 M nitric acid under continuous stirring until a homogeneous mixture was obtained. The mixture was then transferred into a 100 mL Teflon-lined stainless autoclave and the experiment was conducted at 160 °C for 12 h. After heat treatment, the precipitate was separated by centrifugation and re-suspension was done in DI water. The final stage of BiVO_4_/rGO composite was obtained after drying at 70 °C for 24 hrs.

### Characterization

The crystal structure and phase composition of BiVO_4_ compared with composite materials were studied by X-ray diffraction (XRD, Philips X’Pert MPD) using Cu K-alpha radiation. The morphology of the prepared samples were determined using scanning electron microscopy(SEM, JSM-6335F, JEOL) and transmission electron microscopy (TEM,JSM-2010, JEOL). The photoluminescence (PL) spectrophotometer excited with 350 nm was applied in order to analyze the emission wavelength of 500–650 nm (Fluoromax-4 Horiba JobinYvon). The Brunauer-Emmett-Teller (BET) method was used to estimate the surface properties from N_2_ adsorption-desorption isotherm (Adtosorb 1 MP, Quantachrome). The UV–vis spectrophotometer (DRS, Shimadzu, UV–3101PC) with an integrating sphere attachment for diffuse reflectance analysis were used to study the reflectance spectra and further calculate the optical band gap from absorbance data using a Tauc plot of the Kubelka-Munk function^35,36^.

### Photocatalytic activity

Photocatalytic properties of BiVO_4_ in comparison with BiVO_4_/rGO composites were tested over the degradation of methylene blue (MB) aqueous solution (3 ppm, 100 mL) with 0.02 g of photocatalyst. The photocatalytic system were irradiated with halogen lamps (Essential MO, Philips, Thailand) with power of 54 W and light intensity of 145 μW/cm^2^.The mixed suspension between photocatalyst powder and MB solution were stirred for 30 min without light irradiationto ensure that the MB molecules was adsorbed on the catalyst surface. The change in the MB concentration after visible irradiations for 120 min was analyzed from the decrease in absorbance intensity at the wavelength of 664 nm using UV-6100 double beam spectrophotometer (Shanghai Mapada Instruments Co., Ltd).

## Results and Discussion

**Figure 1:**
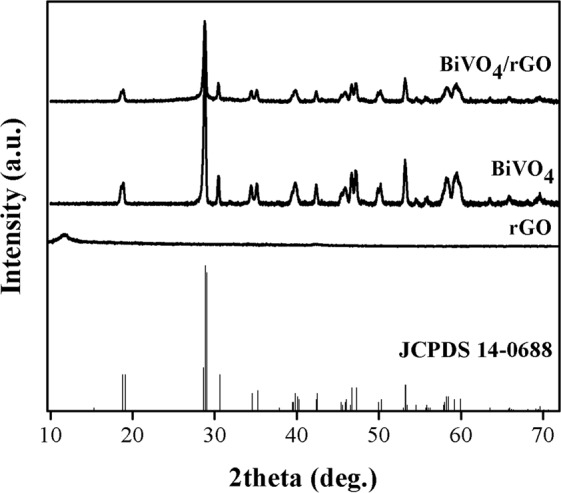
XRD patterns of the rGO, BiVO_4_, and BiVO_4_/rGO composite.

As shown in Fig. 1, the XRD patterns of BiVO_4_, and BiVO_4_/rGO shows that all the diffraction peaks corresponded to the monoclinic phase of BiVO_4_ (JCPDS 14-0688)^37^. In addition, the typical diffraction peak of rGO near 10.8°^38^ were not observed in the XRD pattern of BiVO_4_/rGO composite due to the fact that the addition of rGO in composite sample could yield the stacking disorder of rGO owing to the intercalating of BiVO_4_ into stacked rGO layers, which is in agreement with the literature report of Khalid *et al*.^39^. Also, the introduction of rGO to BiVO_4_ crystal structure might lead to decreasing crystallinity, and result in the broader peaks of BiVO_4_ in composite sample.

**Figure 2:**
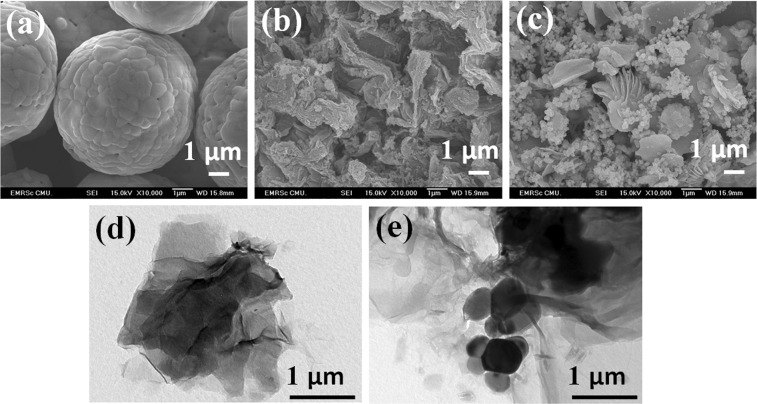
SEM images of (**a**) BiVO_4_ (**b**) rGO, and (**c**) BiVO_4_/rGO composite; and TEM images of (**d**) rGO and (**e**) BiVO_4_/rGO composite.

The morphologies of the prepared samples in Fig. 2(a–c) were separately analyzed by the scanning electron microscope at x1000 magnification. It was found that the BiVO_4_ in Fig. 2(a) constituted the surface roughness of individual spherical particles, which can be obtained in the size ranges of Σ5 μm. In Fig. 2(b), the rGO presented the corrugated structure of mixed-morphology of rGO sheets. In case of BiVO_4_/rGO composite, BiVO_4_ spherical-like particles were uniformly incorporated with rGO sheet, which firmly adhered BiVO_4_ spherical particles outside the surface, as shown in Fig. 2(c). In addition, the smaller particle size with high surface areas of BiVO_4_ was found in the BiVO_4_/rGOcomposite coupled system.The TEM image of rGO sheet in Fig. 2(d) reveals the local wrinkled structure with a thin layer, whereas the TEM images of BiVO4/rGO exhibits a BiVO4 particles attached on a wrinkled surface of rGO.

**Figure 3:**
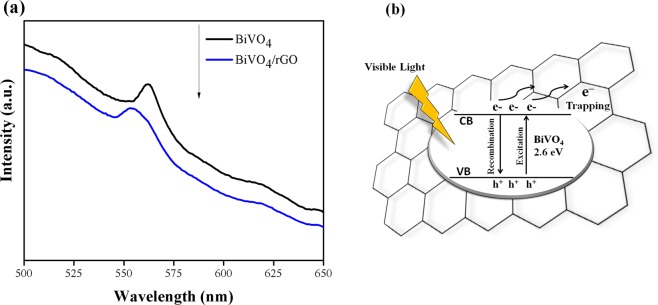
(**a**) Photoluminescence (PL) spectra and (**b**) energy level and electron-hole pair separation/transfer in BiVO_4_/ rGO composites.

Photoluminescence spectroscopy (PL) has been carried out in order to examine the recombination efficiency of photo-induced electrons and holes in photocatalyst. Generally, high emission PL intensity means the rapid charge recombination rate, while a photocatalyst with low PL intensity refers to a low rate of electron/hole pairs. Figure 3(a) shows the PL spectra of BiVO_4_ compared to BiVO_4_/rGO, the PL emission intensity of the latter was the slightly lower intensity corresponding to the lower recombination rate. This improved the separation of electron/hole pairs, and subsequently suppressed the recombination process of BiVO_4_, which is promising for enhancing the photocatalytic activity.

Some part of oxygen-containing functional groups on the surface of rGO disappeared during the phase transformation process (in hydrothermal process), leaving unpaired π electrons on rGO sheets. Thus, rGO can help trapping electron transfering to form π–π electrons, coupling within the aromatic region on rGO surface (see Fig. 3(b)). The results are similar to those reported by Wang *et al*.^40^ and Yang *et al*.^41^ for the enhanced photocatalytic activity of TiO_2_ combined with reduced graphene oxide (rGO).

**Figure 4:**
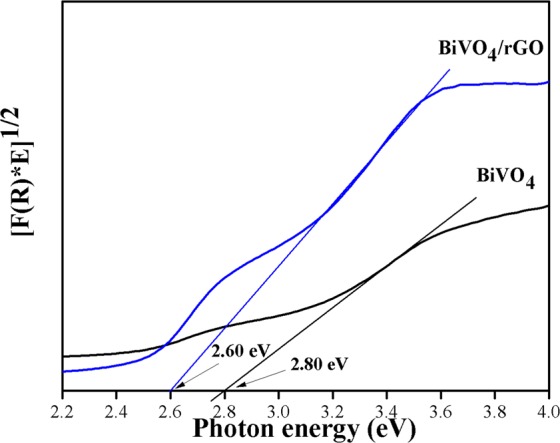
Band gap extrapolation.

Band gap determination in Fig. 4 can be obtained from Tauc’s plot as a function of photon energy (eV) *vs* adsorption multiplied with photon energy. The extrapolation of the straight line in a certain region means that the band gap values was estimated to be Σ2.80 eV and Σ2.60 eV for BiVO_4_ and BiVO_4_/rGO, respectively. The decrease in band-gap possibly linked to the interaction of unpaired π electrons of rGO with free electrons on the BiVO_4_’s surface, playing a significant role in enhancing photocatalytic activity^42,43^.

**Figure 5:**
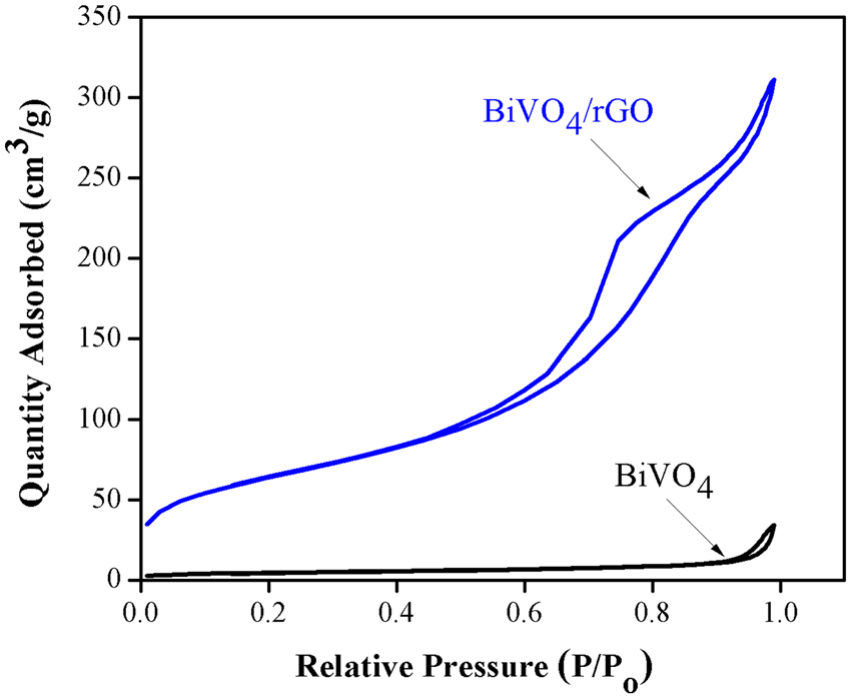
Nitrogen adsorption-desorption isotherm of BiVO_4_ and BiVO_4_/rGO composite.

The surface properties of rGO from our previous work^34^ revealed that the specific surface area of rGO was 1,323.39 m²/g, while the average pore volume and pore diameter are 0.68 cm³/g and 2.06 nm, respectively (See Table 1).The nitrogen adsorption-desorption isotherm of the composite materials compared with BiVO_4_ is revealed in Fig. 5. Both samples presented a typical type IV isotherm characteristic and showed hysteresis loops at the P/P_0_ ranges of 0–1.0, which demonstrated the characteristics of mesoporous materials^44,45^ corresponding to a pore diameter of 8.59 to 17.98 nm for BiVO_4_/rGO and BiVO_4_, respectively. From the hysteresis loops, the adsorbed quantity was found to increase when the rGO was added, leading to an enhanced specific surface area of the composite (228.39 m^2^/g) compared to that in pure BiVO_4_ (16.24 m^2^/g). Thus, the increase in the strength value of the specific surface due to combination of BiVO_4_ with rGO porous material does not only inhibit the electron–hole recombination, but rGO is also beneficial for adsorption to enrich the pollutants around the BiVO_4_ catalyst surface.

**Table 1:** BET specific surface area, pore volume and average pore diameter.

Samples	BET specific surface area (m²/g)	Pore volume (cm^3^/g)	Average pore diameter (nm)
RGO	1,323.39	0.68	2.06
BiVO_4_	16.24	0.05	17.98
BiVO_4_/rGO	228.39	0.47	8.59
Mechanical mixing BiVO_4_/GO	183.36	0.39	8.80

The photocatalytic performance of the pure BiVO_4_ and BiVO_4_/rGO were evaluated by the degradation of model dyes (MB). The degree of MB dye photocatalytic degradation (C_t_/C_0_) was obtained by calculating the change in concentration from the variation of absorbance at the specific wavelength of 664 nm.

**Figure 6:**
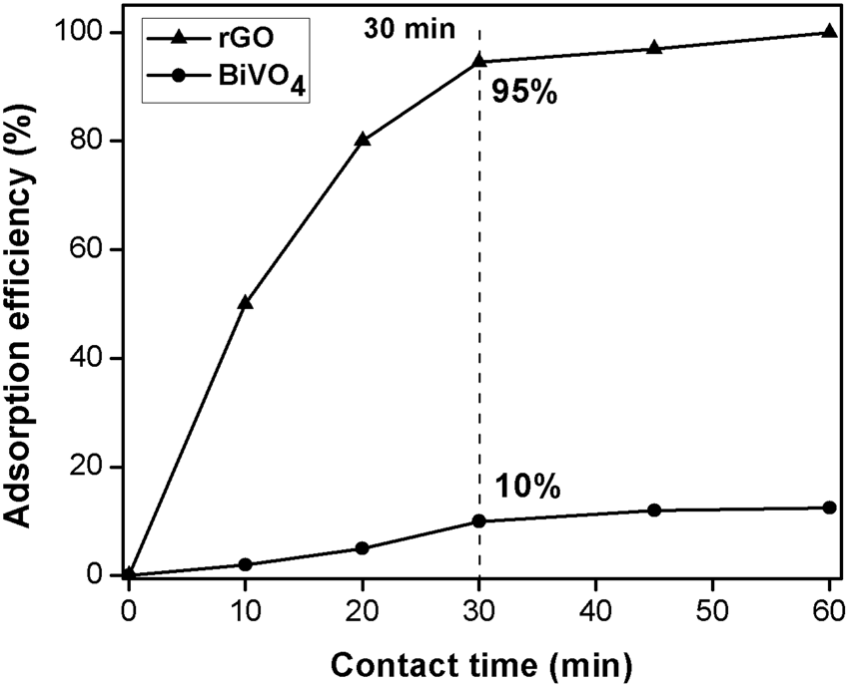
Effect of contact time on dark adsorption of MB on the catalysts.

In order to study the equilibrium contact time, effect of contact time on MB adsorption in dark on BiVO_4_ and GO were studied by varying adsorption times from 10 to 60 min, and the results are illustrated in the Figure below. It was found that the removal of MB rises rapidly along with the contact time and attains the equilibrium after 30 min. The adsorption study was continued further for 60 min but no significant increase was observed in MB adsorption after 30 min contact time. Therefore, 30 min was considered as an equilibrium contact time for dark adsorption (light off) for both of BiVO_4_ and rGO samples (see Fig. 6). Hence, in this study, the remaining experiments for BiVO_4_/GO were carried out and stirred for 30 min under dark to allow adsorption/desorption equilibrium of MB on the catalysts.

**Figure 7:**
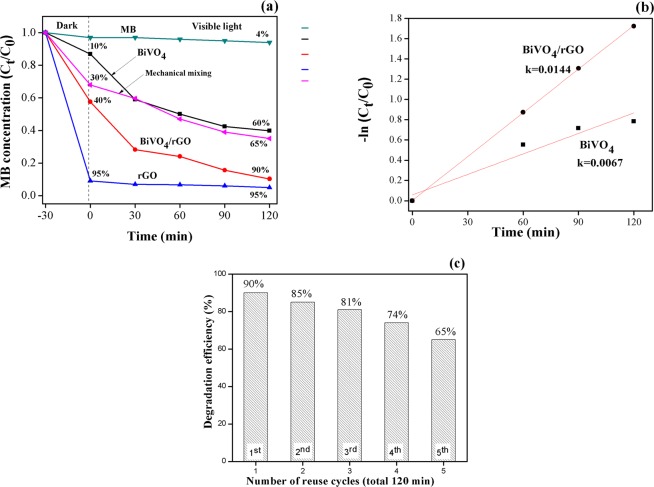
(**a**) C_t_/C_0_ (dark and light on), (**b**) − (ln C_t_/C_0_), and (**c**) reuse of BiVO_4_/rGO composite.

After dark adsorption for 30 min, the photocatalytic degradation efficiency of MB dye reached 10, and 40% for BiVO_4_ and BiVO_4_/rGO, respectively, which is related to the increase in specific surface area obtained from BET results (see Fig. 7(a)). After visible light being on for 120 min, the degradation efficiencyof MB dye was negligible when no photocatalysts were added (about 4%). In case of single phase rGO, the photocatalytic degradation efficiencyof MB reached 90% by adsorption in the dark, but did not further degrade under light irradiation. For pure BiVO_4_, the visible-light photocatalytic performance of MB reached 60% after 120 min. Meanwhile, the degradation efficiency reached 95% when the photocatalysts were replaced by BiVO_4_/rGO.

For the sake of comparison, the mechanical mixed sample of BiVO_4_ and rGO has been prepared and its photocatalytic degradation experiment has been carried out. In a typical process, the as-prepared BiVO_4_ and the rGO were mechanically mixed in the agate mortar. After photocatalytic reaction, the photocatalytic degradation efficiency of the mechanical mixed sample was determined and found to be lower than that of BiVO_4_/rGO prepared by wet chemical process. This might be the result from the decrease of specific surface area of BiVO_4_/rGO via mechanical mixing due to the aggregation of BiVO_4_ particles and also the agglomeration of graphene oxide layers. As shown in Table 1, the specific surface area values measured for mechanical mixed sample amount to 183.36 m^2^/g, which is lower than the BiVO_4_/rGO composite prepared by hydrothermal method (228.39 m^2^/g). In addition, large specific surface area could provide more active sites for the adsorption of pollutants causing further degradation under light illumination.

In order to compare the speed of the photocatalyst under light irradiation, the apparent rate constants (k) were obtained from slopes of the graphs by plotting ln (C_t_/C_0_) versus t. The pseudo-first-order rate constants can be calculated using the following equation^43^:

$$-\mathrm{ln}\,({{\rm{C}}}_{{\rm{t}}}/{{\rm{C}}}_{0})={\rm{kt}}$$

where C_0_ and C_t_ are the initial and remaining concentrations of MB at the different irradiated time (t), respectively. The corresponding pseudo-first-order kinetic plots are shown in Fig. 7(b). The photocatalytic degradation rate constant (k) were 0.0144 and 0.0067 min^–1^for BiVO_4_/rGO and BiVO_4_, respectively, as listed in Table 2.

**Table 2:** Apparent rate constant (k, min^−1^) from the slope of −ln C_t_/C_0_ versus irradiation time.

Sample	Linear regression equation	Rate constant (k, min^−1^)
BiVO_4_	y = 0.0067×	0.0067
BiVO_4_/rGO	y = 0.0144×	0.0144

Moreover, the reusability of BiVO_4_/rGO composite was tested by recovering photocatalyst for multiple cycles, as shown in Fig. 7(c). The result shows that photocatalytic degradation efficiency of MB over BiVO_4_/rGO composite does not significantly differ (Σ5–10%) up to 5^th^ cycle, indicating that the prepared photocatalyst in this study was stable and can be reused up to fifth cycle.

**Figure 8:**
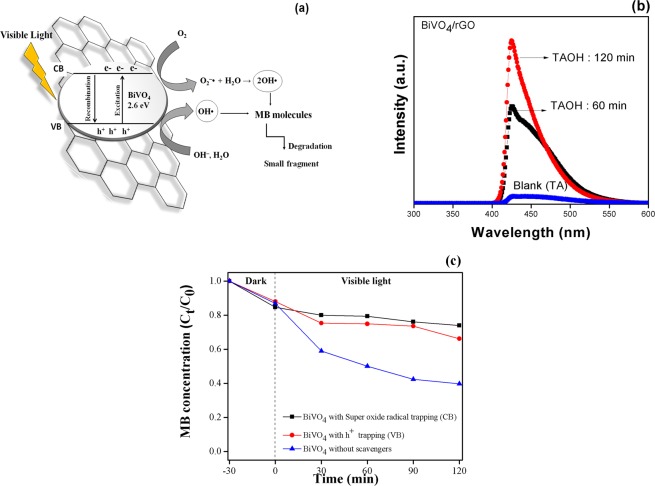
(**a**) proposed mechanism for photocatalytic degradation of MB, (**b**) hydroxyl radical trapping in form of TAOH over BiVO_4_/ rGO, and (**c**) BiVO_4_ p-benzoquinone and ammonium oxalate scavengers.

The proposed mechanism for photocatalytic activity of MB dye over BiVO_4_/rGO composites is shown in Fig. 8(a). Under visible-light irradiation with an appropriate excitation energy, the electrons of BiVO_4_ are excited from the valence band (VB) to the conduction band (CB), thereby, forming the photogenerated electron−hole pairs. The excited electrons in CB of BiVO_4_ can migrate to rGO, and generate OH^•^ via the reduction of O_2_ to yield superoxide radical (O_2_^•*−*^) and hydroxyl radical (OH^•^), which subsequently degrade the MB dye molecules. Meanwhile, the hydroxyl ion (OH^*−*^) adsorbed on the surface can be reduced by the photogenerated holes at VB of BiVO_4_ to give OH^•^ and further react with the target products. Therefore, the BiVO_4_/rGO composites can enhance the photocatalytic activity of BiVO_4_ through the enhanced lifetime of photogenerated electrons/holes and specific surface area.

In order to determine the active species generated by hydroxyl radical (OH^•^) during the photocatalytic process, the terephthalic acid (TA) nonfluorescent substance was introduced as the trapping substance to yield a long lived highly fluorescent 2-hydroxyterephthalic acid (TAOH)^46^. Fluorescence spectra of a TAOH solution generated by BiVO_4_/rGO is represented in Fig. 8(b). The emission intensity at 425 nm (excited by 315 nm) increases with the increased irradiation time from 60 to 120 min, which corresponded to the higher photodegradation efficiency over irradiation time. Also, the oxidation of TA to TAOH confirms the OH^•^ generation during photocatalysis as well as indicate the successful transfer of charge carrier and separation.

In order to prove the photocatalytic mechanism that the photocatalytic reactions happen based on the hydroxyl radical generated via VB or CB, the active species trapping experiment was conducted. In the typical process as reported in literatures^47–49^, p-benzoquinone and ammonium oxalate with concentration of 3 ppm were added to the photocatalytic reaction as O_2_^•*−*^ (CB) and h^+^ (VB) scavengers, respectively. Photocatalytic degradation of MB in the presence of different scavengers over the BiVO_4_ photocatalyst was presented in Fig. 8(c). The results showed that the addition of h^+^ (VB) and O_2_^•*−*^ (CB) scavengers inhibited the photocatalytic degradation of MB. It can be concluded that the position of photocatalytic mechanism as well as the OH· generation could be occurred via both of h^+^ at the CB level and O_2_^•*−*^ at the VB position.

## Conclusions

This study aimed to prepare the monoclinic spherical-shaped BiVO_4_ combined with rGO sheets via co-precipitation using the hydrothermal method. The pseudo-first-order rate constant of BiVO_4_/rGO was about two times higher than that of BiVO_4_. The enhancement in visible photocatalytic activity originated from the injection of excited electrons from the CB of BiVO_4._ The π–π electron coupling between the aromatic regions on rGO surface, increased the separation efficiency of photogenerated electron–hole pairs and further generate OH^•^. Thus, rGO in composites do not only help increasing the MB concentration near the surface active site of BiVO_4_ due to its high specific surface area, but also significantly promoting photogenerated charge separation. In addition, the recycling of the BiVO_4_/rGO photocatalyst became possible and can be effectively separated for 5 cycles. The studies of detailed mechanism using terepthalic acid, p-benzoquinone, and ammonium oxalate scavengers confirmed that the hydroxyl radicals are mainly responsible for photocatalytic activity, which produced from the h^+^ (VB) and O_2_^•*−*^ (CB) in photocatalytic degradation of MB. The hydrothermal synthesis of pure BiVO_4_ are recommended for future research as different synthesis routes can cause the different morphology, particle size and crystallization resulting to different photocatalytic activities.

## References

[1] Houas A (2001). Photocatalytic degradation pathway of methylene blue in water. Applied Catalysis B: Environmental.

[2] Arami M, Limaee N, Mahmoodi N, Tabrizi N (2006). Equilibrium and kinetics studies for the adsorption of direct and acid dyes from aqueous solution by soy meal hull. Journal of Hazardous Materials.

[3] Yang W, Wu D, Fu R (2008). Effect of surface chemistry on the adsorption of basic dyes on carbon aerogels. Colloids and Surfaces A: Physicochemical and Engineering Aspects.

[4] Mathivanan M, Elumalai SS (2017). Moringa oleifera: A cost effective coagulant for dye degradation. Rasayan Journal of Chemistry.

[5] Buscio V, Brosillon S, Mendret J, Crespi M, Gutiérrez-Bouzán C (2015). Photocatalytic membrane reactor for the removal of C.I. disperse red 73. Materials.

[6] Ito T, Adachi Y, Yamanashi Y, Shimada Y (2016). Long-term natural remediation process in textile dye-polluted river sediment driven by bacterial community changes. Water Research.

[7] Thiruvenkatachari R, Vigneswaran S, Moon S (2008). A review on UV/TiO_2_ photocatalytic oxidation process. Korean Journal of Chemical Engineering.

[8] Mishra NS (2017). A review on advanced oxidation processes for effective water treatment, Current World. Environment.

[9] Nickheslat, A., Amin, M. M., Izanloo, H., Fatehizadeh, A. & Mousav, S. M., Phenol photocatalytic degradation by advanced oxidation process under ultraviolet radiation using titanium dioxideม Journal of Environmental and Public Health **2013** 1–9 (2013) 10.1155/2013/815310 PMC3655584 23710198

[10] Lee S, Park S (2013). TiO_2_ photocatalyst for water treatment applications. Journal of Industrial and Engineering Chemistry.

[11] Channei, D., Inceesungvorn, B., Wetchakun, N., Ukritnukun, S. & Nattestad, A. Photocatalytic degradation of methyl orange by CeO_2_ and Fe–doped CeO_2_ films under visible light irradiation Scientific reports **4**, 5757 (1–7) (2004) 10.1038/srep05757 PMC5385822 25169653

[12] Ong CB, Ng LY, Mohammad AW (2018). A review of ZnO nanoparticles as solar photocatalysts: Synthesis, mechanisms and applications. Renewable and Sustainable Energy Reviews.

[13] Dong P, Hou G, Xi X, Shao R, Dong F (2017). WO_3_-based photocatalysts: morphology control, activity enhancement and multifunctional applications. Environmental Science: Nano.

[14] Malathi A, Madhavan J, Ashokkumar M, Arunachalam P (2018). A review on BiVO_4_ photocatalyst: Activity enhancement methods for solar photocatalytic applications. Applied Catalysis A: General.

[15] Hlophe PV, Mahlalela LC, Dlamini LN (2019). A composite of platelet-like orientated BiVO_4_ fused with MIL-125(Ti): Synthesis and characterization. Scientific Reports.

[16] Li H, Yu H, Quan X, Chen S, Zhao H (2015). Improved photocatalytic performance of heterojunction by controlling the contact facet: High electron transfer capacity between TiO_2_ and the {110} facet of BiVO_4_ caused by suitable energy band alignment. Advanced Functional Materials.

[17] Kim Y (2015). Hydrogen evolution: Hybrid Z‐scheme using photosystem I and BiVO_4_ for hydrogen production. Advanced Functional Materials.

[18] Wetchakun N (2012). BiVO_4_/CeO_2_ nanocomposites with high visible-light-induced photocatalytic activity. ACS Applied Materials & Interfaces.

[19] Liu H, Hou H, Gao F, Yao X, Yang W (2016). Tailored fabrication of thoroughly mesoporous BiVO_4_ nanofibers and their visible-light photocatalytic activities. ACS Applied Materials & Interfaces.

[20] Sharma M, Behl K, Nigam S, Joshi M (2018). TiO_2_-RGO nanocomposite for photocatalysis and environmental applications: A green synthesis approach. Vacuum.

[21] Lv S, Wan J, Shen Y, Hu Z (2017). Preparation of superlong TiO_2_ nanotubes and reduced reduced graphene oxide composite photocatalysts with enhanced photocatalytic performance under visible light irradiation. Journal of Materials Science: Materials in Electronics.

[22] Li X (2018). Reduced graphene oxide enhanced amine-functionalized titanium metal organic framework for visible-light-driven photocatalytic oxidation of gaseous pollutants. Applied Catalysis B: Environmental.

[23] Liu X, Cai L (2019). A novel double Z-scheme BiOBr-RGO-polyaniline photocatalyst: Study on the excellent photocatalytic performance and photocatalytic mechanism. Applied Surface Science.

[24] Wang Q (2019). Enhanced photocatalytic degradation and antibacterial performance by RGO/CN/BiOI composites under LED light. Applied Surface Science.

[25] Ghouri ZK, Elsaid K, Abdala A, Al-Meer S, Barakat NAM (2018). Surfactant/organic solvent free single-step engineering of hybrid graphene-Pt/TiO_2_ nanostructure: Efficient photocatalytic system for the treatment of wastewater coming from textile industries. Scientific Reports.

[26] Xu Y, Li Y, Wang P, Wang X, Yu H (2018). Highly efficient dual cocatalyst-modified TiO_2_ photocatalyst: RGO as electron-transfer mediator and MoSx as H_2_-evolution active site. Applied Surface Science.

[27] Wang X, Zhao X, Zhang D, Li G, Li H (2018). Microwave irradiation induced UIO-66-NH_2_ anchored on graphene with high activity for photocatalytic reduction of CO_2_. Applied Catalysis B: Environmental.

[28] Yu H, Xiao P, Tian J, Wang F, Yu J (2016). Phenylamine-Functionalized rGO/TiO_2_ photocatalysts: Spatially separated adsorption sites and tunable photocatalytic selectivity. ACS Applied Materials & Interfaces.

[29] Lonkar SP, Pillai VV, Alhassan SM (2018). Facile and scalable production of heterostructured ZnS-ZnO/Graphene nano-photocatalysts for environmental remediation. Scientific Reports.

[30] Liu B, Zhenhua W, Zhou S, He J (2015). Synthesis and characterization of a novel BiVO_4_/SiO_2_ nanocomposites. Materials Letters.

[31] Fang D (2017). BiVO_4_-rGO with a novel structure on steel fabric used as high-performance photocatalysts. Scientific Reports.

[32] Channei D, Nakaruk A, Khanitchaidecha W, Jannoey P, Phanichphant S (2018). Adsorption and photocatalytic processes of Mesoporous SiO_2_-Coated Monoclinic BiVO_4_. Frontiers in chemistry.

[33] Strobel R, Metz HJ, Pratsinis SE (2008). Brilliant yellow, transparent pure, and SiO_2_-coated BiVO_4_ nanoparticles made in flames. Chemistry of Materials.

[34] Channei D, Nakaruk A, Phanichphant S (2018). Controlled oxidative ageing time of graphite/graphite oxide to reduced graphene oxide in aqueous media. Journal of the Australian Ceramic Society.

[35] Murphy AB (2007). Band-gap determination from diffuse reflectance measurements of semiconductor films, and application to photoelectrochemical water-splitting. Solar Energy Materials & Solar Cells.

[36] Muniz EC (2011). Synthesis and characterization of mesoporous TiO2 nanostructured films prepared by a modified sol–gel method for application in dye solar cells. Ceramics International.

[37] Ma Y, Jiang H, Zhang X, Xing J, Guan Y (2014). Synthesis of hierarchical m-BiVO_4_ particles via hydro-solvothermal method and their photocatalytic properties. Ceramics International.

[38] Sampath S (2013). Direct exfoliation of graphite to graphene in aqueous Mmedia with diazaperopyrenium dications. Advanced Materials.

[39] Khalid NR, Ahmed E, Hong Z, Sana L, Ahmed M (2012). Enhanced photocatalytic activity of grapheme-TiO_2_ composite under visible light irradiation. Current Applied Physics.

[40] Wang H (2019). Facile prepared ball-like TiO_2_ at RGO composites for oxytetracycline removal under solar and visible lights. Water Research.

[41] Yang WD, Li YR, Lee YC (2016). Synthesis of r-RGO/TiO_2_ composites via the UV assisted photocatalytic reduction of graphene oxide. Applied Surface Science.

[42] Khannam SKDM, Sharma S, Dolui S (2016). A reduced graphene oxide incorporated TiO_2_ photoanode for high efficiency quasi solid state dye sensitized solar cells based on a poly-vinyl alcohol gel electrolyte. RSC Advances.

[43] Li L, Yu L, Lin Z, Yang G (2016). Reduced TiO_2_-reduced graphene oxide heterostructure as broad spectrum-driven efficient water-splitting photocatalysts. ACS Applied Materials & Interfaces.

[44] Sotomayor FJ, Cychosz KA, Thommes M (2018). Characterization of micro/mesoporous materials by physisorption: Concepts and case studies. Accounts of Materials & Surface Research.

[45] Xue C (2017). Fluoride doped SrTiO_3_/TiO_2_ nanotube arrays with a double layer walled structure for enhanced photocatalytic properties and bioactivity. RSC Advances.

[46] Ishibashi KI, Fujishima A, Watanabe T, Hashimoto K (2000). Detection of active oxidative species in TiO_2_ photocatalysis using the fluorescence technique. Electrochemistry Communications.

[47] Rodríguez EM, Márquez G, Tena M, Álvarez PM, Beltrán FJ (2015). Determination of main species involved in the first steps of TiO_2_ photocatalytic degradation of organics with the use of scavengers: The case of ofloxacin. Applied Catalysis B: Environmental.

[48] Zhang Z (2018). Facile one-step synthesis of TiO_2_/Ag/SnO_2_ ternary heterostructures with enhanced visible light photocatalytic activity. Scientific Reports.

[49] Qi. Zhang N (2016). Advanced Fabrication of Chemically Bonded Graphene/TiO_2_ Continuous Fibers with Enhanced Broadband Photocatalytic Properties and Involved Mechanisms Exploration. Scientific Reports.

